# American Medical Association

**Published:** 1856-06

**Authors:** 


### AMERICAN MEDICAL ASSOCIATION.

The Ninth Annual Meeting of the Association, was held in the city of Detroit, Mich., in Fireman's Hall. The Session was opened on Tuesday, May 6th, at 11 o'clock A. M. The president, Dr. Geo. B. Wood of Pennsylvania, in the chair.

Dr. Pitcher, of Michigan on behalf of the Committee of Arrangements, offered, in a few eloquent remarks, a cordial welcome to the delegates.

The list of Delegates who had registered their names, was reported by the Committee of Arrangements.

The roll was then called by Dr. Wister, of Pennsylvania.

On motion of Dr. Thomson, of Delaware, a recess of fifteen minutes was taken to allow the delegates from the respective States to report one member from each State represented, as a committee to nominate officers for the ensuing year.

At the expiration of the recess, the Association was called to order, and the different State delegations then reported their choice, respectively, of a delegate to serve on the nominating committee, which was constituted as follows:

*Maine*—N. P. Monroe.
*New Hampshire*—H. Peirce.
*Massachusetts*—H. H. Childs.
*Vermont*—C. L. Allen.
*Rhode Island*—J. E. Warren.
*Connecticut*—David Harrison.
*New York*—William Rockwell.
*New Jersey*—L. A. Smith.
*Pennsylvania*—John Neill.
*Delaware*—J. W. Thomson.
*Maryland*—P. Wroth.
*South Carolina*—E. Geddings.
*Tennessee*—J. B. Lindsley.

*Kentucky*—W. S. Sutton.

*Minnesota*—C. W. LeBoutilier.

*Michigan*—M. Gunn.

*Ohio*—Thos. W. Gordon.

*Indiana*—Dr. Winton.

*Illinois*—H. Noble.

*Wisconsin*—W. H. Brisbane.

After the Nominating Committee had retired, Dr. Pitcher, of Michigan, from the Committee of Arrangements, submitted the following report:

In conformity to the domestic and social usages of the place of meeting, the committee have to suggest that the sessions of the Association take place in accordance with the following plan, and that they commence and terminate each day at the hours designated therein:

*Tuesday*—Morning session begins at 9 A. M. and ends at half-past 12 M. Afternoon session begins at 2 P. M. and ends at 5 P. M.

*Wednesday*—Morning session begins at 9 A. M. and ends at half-past 12 M. Afternoon no session.

*Thursday*—Morning session begins at 9 A. M. and ends at half-past 12 M. Afternoon session begins at 2 and ends at 5 P. M.

*Friday*—Morning session begins at 9 o'clock A. M.

This arrangement of the hours of meeting and adjournment conforms, also, to the suggestions contained in the resolutions of Dr. N. S. Davis of Illinois, and which were, on his motion, referred to this Committee for their consideration by a vote of the Association, (see vol. viii. p. ). Regard for the mover of the resolutions, and the authority of the body by which they were submitted to us, requires from the Committee a respectful reply. Your committee, in view of the existing state of our professional literature, feel reluctant to advise a departure from the present mode of laboring to promote a higher degree of culture in those preparing to become members of the medical profession, and to establish in those already engaged in its duties a habit of recording the results of their observations. They think that the effects of such a change as is contemplated in the resolutions of Professor Davis, and the more amplified expression of his idea, contained in the address of the then President, Dr. Pope, of Missouri, delivered at Philadelphia, in 1855, can be easily foreseen. To a few who are gifted with colloquial powers, and to others who have undergone the discipline required to fit them for public debate, the interest of the meetings conducted upon the plan proposed in the resolutions would be greatly increased, but as the great body of the Association would, voluntarily, it is true, be excluded from participation in these exercises, the enthusiasm which now characterizes our anniversaries would subside, and with it the professional *esprit du corps* which has already been developed through the instrumentality of the Association. We presume that the objects for which this organization was effected have not been lost sight of by the majority of its members. Neither can it be pretended that those purposes have been so far accomplished as to justify us in laying it aside, or of diverting it from its original design.

Your committee feel that the profession has no right to rail at the public for misappreciation of it, so long as we continue to admit men into its folds destitute of that knowledge, both in nature and degree, necessary to make a decent appearance in general society, or to fit a man for the more ordinary and less responsible pursuits of life. From the early records of the Association it appears that this conviction on the part of the profession in the United States, connected with the design of reforming, in certain particulars, the medical schools of our country, led to its organization in 1847, and until its mission in both respects has been accomplished, the committee would reluctantly recommend the adoption of any measure tending in their judgment to divert it from the design of its creation. Thus far the influence of the Association has gradually extended itself into the rank and file of the profession. It has increased the number of writers, given an impulse to the medical mind, and encouraged a useful and laborious class, gratified to observe, and willing to submit their observations to the public, because they can be incorporated into the body of the Transactions without being subjeced to a sifting criticism. It is true, that in this way articles have been printed that did not always enure to the credit of the Association, but at the same time, and by that means, motion and fertility have been given to minds that would have lain fallow and unproductive, which the dread of the conspicuity belonging to a mental gymnasium would have driven into deeper obscurity. The committee, however, whilst they would resist any tendency to radicalism in their own opinions, cannot dismiss the subject without expressing their belief that, in order to secure the objects of our organization, it is as necessary to increase the depth and breadth of its base as to elevate the shaft designed to spring from it, for without such preparation, the superstructure, however beautiful in aspect, would be of transient duration.

Having arranged the hours for meeting and adjourning, so as to place it in the power of the Association to adopt or reject, without inconvenience, the proposition of Dr. Davis, the committee respectfully ask to be excused from submitting a distinct proposition on the subject.

By order of the Committee of Arrangements,

Z. Pitcher, Chairman.

The report was accepted.

The President announced the death of one of the ex-presidents of the Association, Dr. John C. Warren, of Boston, Massachusetts.

Dr. Childs, of Mass., felt compelled to say a few words in this connection. He had been associated with the deceased for more than half a century, and should feel that he had been derelict of duty if he neglected to speak in his laudation. Dr. Warren was the nephew of Joseph Warren, who fell gloriously at the battle of Bunker Hill. He was at the head of his profession in Massachusetts—had been President of the State Medical Society, and occupant of other elevated medical positions. His professional reputation was high, and his personal reputation spotless. His fame was not confined to Massachusetts. Though devoted to medical science, he was not limited to that alone, but paid attention to every branch of literature and art. If young members of the profession would be useful and eminent, they should follow the example of Dr. J. C. Warren. To the older, the speaker would point out Dr. W.'s moral character as an exemplar. Such a life as his inevitably terminates in a death beautified by a surety of eternal happiness.

Dr. Gross, of Kentucky, made some remarks eulogistic of the deceased. He alluded to his high reputation—a reputation, he observed, not confined to America, but extending to every corner of the civilized world. Dr. Warren was the Nestor of American surgery. Dr. G. concluded by offering the following:

*Resolved,* That a committee of five be appointed to draft resolutions expressive of the feelings of this Association at the loss of their late associate, Dr. John C. Warren.

The resolution was adopted, and the President appointed as such committee, Dr. Gross, of Kentucky, Dr. Childs of Massachusetts, Dr. Wood of New York, Dr. Pitcher of Michigan, and Dr. Geddings of South Carolina.

On motion, the Association adjourned to 2 P. M.

*May 6th—Afternoon Session.*

The Association was called to order at 2 o'clock P. M.

The Secretary read letters from the State Medical Society of Tennessee, and from the University of Nashville, inviting the Association to hold its next annual session at Nashville, Tennessee. Also one tendering the use of the Hall of Representatives of that State for the purposes of said session.

On motion of Dr. Brodie, of Michigan, referred to Committee on Nominations.

The Committee on Nominations submitted the following report.

The Committee on Nominations unanimously nominate the following officers of the American Medical Association for the ensuing year:

*President*—Dr. Zina Pitcher, of Detroit.

*Vice Presidents*—Drs. Thomas W. Blatchford, of New York; Wm. K. Bowling, of Tennessee; E. Geddings, of South Carolina; W. H. Brisbane, of Wisconsin.

*Secretaries*—Drs. Wm. Brodie, of Michigan; R. C. Foster of Tennessee.

*Treasurer*—Dr. Caspar Wister, of Pennsylvania.

The report was accepted, and the nominations unanimously confirmed.

On motion of Dr. Atlee, of Pennsylvania, the President delivered his annual address.

### ADDRESS.

Custom demands, as one of the expiring duties of your presiding officer, that he should leave a legacy at least of good wishes, if not of something more valuable behind him. In compliance with this duty, I propose to say a few parting words, which, whatever else they may convey to you, will assuredly not interpret duly the sentiments of him who utters them, unless they make you sensible of his grateful and most kindly feelings towards his fellow members, and of his zealous interest in the great objects of our Association.

The present is a suitable occasion for taking a survey of the Association; for looking around towards the boundaries of its labors, interests, and duties, and noting whether something may not present itself in the view, which may profitably occupy, for a few minutes, our serious and earnest attention. Let us first throw a comparative glance from the present backward to the past. Perhaps by so doing we may be better prepared to look forward intelligently into the future.

Have the hopes with which the Association set out in its mission of self imposed duty, been fulfilled? Has the loud call which it sent forth through the nation, startling the profession from its uneasy slumber, succeeded in awakening it thoroughly to a sense of its high responsibilities, and arousing a determined spirit of progress? Or has it died away in gradually diminishing echoes, leaving but a drowsy memory of that spirit-stirring appeal? Have the annual gatherings of the elect of the profession, their joint deliberations in council, their various legislation, the practical inquiry set on foot or encouraged, not omitting their exploits at the festal board, and kindly interchange of thought and sentiment in social assemblage; have all these been without fruit? Have they been the mere course of a phantom ship through the ocean of human events, leaving no track in its passage, and bearing no freight onward to its destination?

Were we to listen to the clamours of opposition, the whisperings of discontent, or the murmured disappointment of an over-excited expectation, we might be disposed to give to these questions an unfavorable answer; to cease our struggles for an unattainable good; and with the wings of the spirit folded, and its head drooping, to submit in sadness to an inexorable destiny, chaining us in submission to all present evils, and jealous even of a glance towards the higher and the better.

But happily, such is not the voice of a clear and unbiassed judgment. It is true that the Association has not accomplished the whole of what it aimed at. Like all other young things, conscious of a stirring life within, and feeling no limits to its yet untried powers, it hoped and strove beyond the possible; it struck in its soaring flight against the iron will of circumstance, and for a time, at least, fell back, stunned though not crushed, into humbler aims. Yet, even as regards medical education, which is the main point of failure, its efforts have not been all thrown away. Some advance, however small, has, I think, been already made; and bread, moreover, has been cast upon the waters, to be found after many days.

But outside of this vexed subject, much, very much has been accomplished. I will not appeal to the ponderous volumes of our Transactions. They speak for themselves. To say that there is no chaff among their solid contents, would be to say what is neither now nor ever has been true of any large book, with one solitary exception. But I believe that all present will join me in the opinion, that one who searches these records, with a sincere and candid spirit, will find in them much that is good; much that may warrant the self-congratulation of the Association for having originated, or called it forth.

But, whatever credit may be given to these living witnesses of our labors, one fact is evident, that the medical mind has been aroused; that the spirit of improvement has breathed upon the masses of the profession, and everywhere scattered germs, which are now developing, and will probably hereafter continue to develop, even in a still higher ratio, into earnest efforts for self-culture, and general advancement.

Stagnation, in the moral as in the physical world, generates corruption. Agitation, though often in its extremes a cause of evil, and sometimes of unspeakable present wretchedness, generally purifies in the end, and, if restrained within due limits, is a source of unmixed good. The medical mind, anterior to the birth of this Association, was in a state of comparative inertia. In all the departments of the profession, the educational as well as the practical, material interests began to predominate. There was danger that the profession might sink to the level of a mere business. Noble aims; high aspirations; the general good; the spirit of self-sacrifice; these began to be looked on as wordy inflations. The great struggle seemed to be, in the teaching department to gather pupils; in the practical, to gather patients; in both, to swell the pockets. Stagnation of the professional spirit was breeding noxious influence in its motionless depths. No wonder that quackery loomed upward, as regular medicine began to sink. There was danger that the public might be able to see little difference between them; and the fact is, that the line of demarcation was not very distinct, even to the professional eye. They ran into each other, at their extremes, by quite insensible shades.

But the Association arose, and a new spirit was awakened. Many had been watching this apparent abasement of the profession with sorrow; but they were powerless in their isolation. No sooner had the flag of the Association been given to the breeze, than they hastened to join its standard. From all quarters, and from the remotest bounds of the country, volunteers poured in to join this great crusade against the evils which had been usurping the sacred places of the profession. The mass of medical society was moved to its very depths. Hundreds upon hundreds came forth from their sheltering privacy, and threw their souls into the grand movement which was to reconquer, to purify, and regenerate the prostrated glory of their calling. The feeble voice of opposition was heard for a moment; but was soon drowned in the overwhelming shouts of the masses, crying out, Onward! Onward! Even the advocates of the material principle, who could not raise their souls above the level of dollars and cents, found it expedient to chime in for a time with the almost universal voice; and to the enthusiastic it seemed as though a professional millenium was approaching. I need not follow the march of the crusade. I need not recal the varied experience which has but confirmed that of all other revolutionary uprisings, that, except under the influence of a power higher than human, which can regenerate the hearts of men, whatever temporary change may be made in the surface of things, in mere form and arrangement, it is only by the slow working of time that radical and lasting reforms can be effected. Who ever beheld a great nation made by a written constitution? We have had paper republics as thick as the leaves in Vallombrosa; but where, and what are they now? To make a great and free nation, the people must have the principles of greatness and freedom implanted in their hearts. So is it with lesser Associations. It is vain to alter forms, unless the substance is altered too. The Association has discovered this truth. It no longer seeks to work miracles, but is content with following the methods of nature and providence. It has done a great thing in beginning the movement. It is doing what it can to further that movement, and to consolidate its results.

Who is there that has lived and observed through the last ten or fifteen years, who cannot see that our profession has been moving onward and upward since its great awakening; perhaps slowly, perhaps now and then halting, but on the whole advanciug, and with an irresistible force, because it is that of the mass. It is not now a few leaders who are kindling by their own enthusiasm a feeble and temporary blaze of excitement in the multitude; dragging them forward as with cords by their own strong zeal and fiery spirit; it is the inborn soul which is animating the great body, and carrying it forward in its legitimate course.

Had the Association done nothing else, I will not say than originating, but even than aiding and concentrating this rising up of the profession, it would have performed a service entitling it to everlasting gratitude, and to an imperishable name in the medical annals of our country.

A great benefit conferred on the profession by the Association, was the preparation and adoption of a code of medical ethics. I need not say to *you*, that this code is merely an expression of the great principles of truth, justice and honor, in their application to the relations of physicians to one another, their patients, and the public. It is the voice of wisdom and experience speaking from the past, and meets a ready response in the breast of every man possessed of a good heart, a sound judgment, and correct moral principle. Should any one find a repugnance to the observance of its rules rising up within him, let him for a moment reflect, whether this may not spring from some evil source in himself; whether it may not be the result rather of an unwillingness to make what he may deem a sacrifice at their suggestion, than of a real conviction of their injustice or impropriety. Which is more likely to be true; the unbiassed and unselfish judgment of the wisest and most experienced in the profession, or an individual decision which may at least be suspected of a selfish basis, and of which no man, if his interests or feelings are in any degree involved, can say that it is quite pure; for no man can judge impartially in his own case. A becoming modesty would lead him to suspect that the fault might be in himself, and a becoming spirit to search into the depths of his own heart for the root of the evil, and to pluck it out if discovered. I have no doubt that a full observance of these rules would tend more than any one thing else, to maintain harmony in the profession, and to elevate it in the public esteem. It would render impossible those unseemly disputes, founded on petty jealousies, and supposed opposition of interests, which, probably beyond any other single cause, expose the profession to obloquy and ridicule. A copy of the Code should be placed in the hands of every young man about to enter upon the practice of medicine, with the urgent advice that he should make it the guide of his professional life; that he should not only regulate his conduct in conformity with its precepts, but should educate his heart into a real preference for them. Would it not be an object worthy of the attention of the Association to provide for such a distribution; at least, by the publication of a large edition of the Code, to put it in the power of individuals or societies, who might be disposed to engage in this work of beneficence, to do so with as little cost to themselves as possible? I do honestly believe that, to a young physician going forth into a life full of moral conflicts, the wearing of this ægis would be one of his surest defences; that, next to the holy scriptures, and the grace of God, it would serve most effectually to guard him from evil.

Not one of the least advantages of the Association is that, representing as it may be said to do, the medical profession of the country, its voice, when nearly or quite unanimous, will be considered as that of the whole medical body, and thus have weight both in the community at large, and in the legislative councils of the nation. It is only thus that the profession can make their special opinions and wishes known and felt. I have been told that the representations of the Association had much weight in determining a satisfactory arrangement of the question respecting the relative rank of the Surgeons in the navy. It is to be presumed that the patriotic physician who brought before Congress the memorable measure for establishing a general inspection of imported drugs, was materially aided in carrying it through by the approving voice of the profession, speaking in the memorial from this body. On another occasion, you were heard, through your resolutions, pleading in the Halls of Congress in favour of a great measure of honesty and justice, when you petitioned for an international copyright law between the United States and Great Britain; and, should such a law ever be passed, it will not be claiming too much for the Association to say that it will have contributed to that result. Your resolutions, from time to time, in advocacy of a system of registration of births, deaths, &c., have probably also added something to the mass of influence which has brought legislation to bear on this most important subject, though, it must be acknowledged, hitherto but very partially, and, with some honorable exceptions, ineffectually.

There is one other view of the beneficial influence of our great gatherings which I cannot pass unnoticed.

The effect of isolation is well known in breeding excessive self-respect, distrust of others, and narrow, selfish, and sectional views and feelings. Man is naturally gregarious; and it is only in association that his nature can receive its full development; that the seeds of the better qualities within him can be made to germinate, and the qualities themselves to grow up, under culture, into their just magnitude and proportions.

Our Association brings together many who would otherwise never meet, from sections remote from each other, and differing much in views, habits, and feelings. We come, partly at least, for relaxation from the cares and toils of business, prepared and desirous to be pleased. Each one naturally, and without design, turns out the fairest side of his character, "his silver lining to the sun;" and all consequently make and receive favourable and kindly impressions. Each place selected for our meetings feels its character for hospitality involved in the reception of its guests, and every effort is made to extend all proper courtesies and kindnesses to the assembled representatives of the profession. In parting, therefore, we carry with us friendly remembrances of one another, and of the place of assemblage, to our several far separated homes. These remembrances serve as so many cords not only to bind the members of the profession together in one harmonious whole, but also, intertwined with other similar agencies, to counteract the centrifugal tendencies of our political system, and to keep it moving onward, each part in its due place, in that majestic course which, while shedding beneficent influences throughout its own great circle, attracts the admiring and hopeful gaze of humanity everywhere.

Having thus hastily scanned the present and past of the Association, let us turn our thoughts briefly towards the future. A few words will convey all that I have to address to your attention.

It seems to me that experience should have taught us this one lesson; not to aim at once at sweeping changes; but, having determined what great objects are desirable, to keep these always in view, and, by the persevering use of such influences as may be at our command, securing one point in advance before hastening to another, to move on slowly but steadily to our ends. These must ever be the improvement of the profession itself, the advancement of medical science, and the promotion of the public good, so far as that may, in any degree, be connected with our special pursuit. Each of these three points requires a brief notice.

In the improvement of the profession, the Association has from its foundation recognized, as an essential element of success, a higher degree of qualification in those who are to become its members. But for the attainment of this object they can use no coercive measures. The only power they can exercise is that of opinion. Our only appeal is to the judgment and conscience of those concerned. But much may in time be done in this way. It is impossible that intelligent and honorable individuals, possessed of that share of conscientiousness which belongs to most men, and is certainly not deficient in our profession, should long resist such appeals, proceeding from a source so worthy of respect as this. Let us reiterate, from time to time, our convictions of the necessity for improved preparatory education, for a longer devotion to the proper studies of the profession, for a junction of clinical with didactic instruction, and finally for something more than a mere nominal examination before admission to the honour of the doctorate, or the privileges of a license to practice; points which have ever been insisted on by the Association; let us, I say, reiterate these convictions; and like slowly dropping water, they will at length, however gradually, wear their way through the hardest incrustation of prejudice, interest, indolence, or indifference, and reach the conscience with irresistible effect.

While bringing to bear upon this resistance, the considerations of reason, duty, honour, and even an enlightened self-interest, we must carefully avoid all violence of procedure, as likely only to add the hostility of passion to other opposing influences. By this course universal opinion will be gradually conciliated; and interest itself will find its own ends best promoted by compliance with the general will. Already some advance has been gained in this direction; and the Association, by perseverance may yet see all its reasonable wishes accomplished.

In relation to other measures for elevating the character and increasing the efficiency of the profession, there appears to me nothing more at present for the Association to do, than to go on as it has begun. Its continued existence alone is a great good; for it is annually bringing large numbers, simply through membership in its body, to participate in its feelings, and to acknowledge its obligations. Let us then maintain unshrinkingly the standard of professional honour and morals that we have erected, and decline association with those who will not recognize that standard, or having recognized, abandon it. Let us adhere unswervingly to the line which has been drawn between regular and irregular medicine, and treat the practitioners of the latter with the silent disregard they merit. This is the only course for the regular practitioner. To wage a war of words with quackery, is to do what it most delights in. It would be to contend, under the government of honour and principle, with antagonists who acknowledge no such restraints. In our private intercourse with friends and patients, we may explain the grounds of difference between ourselves and the irregulars, may demonstrate the absurdity of their pretensions, the danger of their practice, and the iniquity of their conduct; in short, may endeavour to enlighten wherever light is acceptable, or can penetrate. We may even, if the public interest seem to require it, put forth refutations of false doctrine and assertion, and exposure of subterfuge, trickery, and imposture; but with the irregulars themselves we should enter into no relation, whether of friendship or hostility. I do not say that there may not be honourable and honest, though ignorant or bewildered men among them. But we cannot discriminate. With the presumed advantages of their association, they must be content to take also the disgrace.

There is a point to which I would call the attention of the members of the Association individually. We have been called *Allopathists,* in contradistinction to a sect of irregular practitioners who have taken to themselves the title of *Homœopathists;* the latter term signifying that its professors treat disease by influences similar in their effects to the disease itself; the former that *other*, and of course dissimilar influences are used. It must be remembered, that the designation was not adopted by ourselves, but conferred upon us by Hahnemann and his followers. The intention was obvious. It was to place the regular profession, and their own scheme, upon a similar basis. They practised on one principle, we on a different and somewhat opposite principle. They graciously allowed that our principle was not altogether ineffective; that we did sometimes cure our patients; but theirs was sounder in theory and more successful in practice. Now, by recognizing the name, we necessarily recognize the principle also, and thus put ourselves in a false position. In deciding between them and us, the ignorant masses think they are deciding between two systems, neither of which they understand, but of which they must judge, upon the grounds of relative success. Diseases often get well of themselves, if left alone. The genuine homœopathist leaves them alone, and they often consequently terminate in recovery. This success is magnified by methods well understood; and multitudes are thus led astray, especially among the delicate and refined, who abominate the taste of medicine themselves, and are equally averse to the task of forcing it down the reluctant throats of their children. But we are *not* allopathists. The regular practice of medicine is based on no such dogma, and no exclusive dogma whatever. We profess to be intelligent men, who seek knowledge, in reference to the cure of disease, wherever we can find it, and, in our search, are bound by no other limits than those of truth and honour. We should not hesitate to receive it from the homœopathists, had they any to offer. We would pick it up from the filthiest common-sewer of quackery; for, like the diamond, it has this excellent quality, that no surrounding filth defiles it, and it comes out pure and sparkling, even from the kennel. This is the light in which the medical profession should present itself to the community. We are men who have sought in every possible way to qualify ourselves for the care of their health. We present them, in our diplomas, the evidence that we have gained sufficient knowledge to be trusted with this great charge; and we stand pledged before them to extend our knowledge and increase our skill, as far as may lie in our power. Membership in our honourable profession is the proof we offer, that we are no false pretenders, no interested deceivers; but upright men, intent on the performance of our professional duties. This the people can understand. But when we designate ourselves as *allopathists*, they may well ask, in what, are you better than any other medical sect, than the *homœopathists*, the *hydropathists*, the *Thomsonians*, the *eclectics?* Let us discard, therefore, the false epithet. Let us not only never employ it ourselves, but show that, when applied to us by others, it is inappropriate and offensive, and that the use of it in future would be contrary to gentlemanly courtesy, and the proprieties of cultivated society. I say again, we are not *allopathists;* we are simply *regular practitioners of medicine,* claiming to be honest and honourable—in other words to be gentlemen.

The efficiency of our profession is to be increased not only by increasing its qualifications, but also by all upright measures calculated to win the public confidence, and thus widen the field of our operations. In this respect, I do not know that the Association can do better than to persevere as it has begun; and, by the propriety and dignity with which it conducts its own proceedings, to show to the world the high influences under which the profession acts, and demonstrate that it possesses those qualities of self-government, so useful to the medical practitioner, and so characteristic of the gentleman in all his relations.

The improvement of the *science* of medicine, has always been a favorite object of the Association. The appointment of committees to investigate and report on certain stated subjects, the reception of voluntary communications, the offering of prizes to competing contributors, and the publication of our Transactions annually, are the means employed for this purpose, and I have nothing better to suggest.

The remaining point for consideration, is the promotion of the public good. Happily, such is the nature of our profession, that the more we improve ourselves, the better do we fulfil this great duty. But there is something else to be done. There are certain great interests of the community, relating to their health, of which medical men are the only good judges, and the various influences affecting which, they only can duly appreciate. Upon these points it is our duty to be ever on the watch, and not only like faithful sentinels, to give notice of danger, but, like heaven-appointed agents, as we are, to use our best efforts and influence to prevent or remove it, and, in every practicable way, to guard the public health.

To the establishment of a general system of registration throughout the country, our attention has already been given. We should not relax our efforts, until the great end has been accomplished.

There is another subject deserving of our most serious consideration. You are all aware what advances have recently been made by the small pox in many parts of our country. Thousands are perishing annually, for whose deaths we are, as a profession, in some degree accountable There is no occasion for this mortality. Vaccination and revaccination, duly performed, and under proper circumstances, are, I will not say an absolutely certain, but a very nearly certain safeguard. I have never known of death from small pox, after an efficient revaccination; and only one instance of the occurrence of varioloid. But the profession and the community have both been too careless upon this point. Food for the pestilence has been allowed to accumulate; and it has been rioting with fearful results in many parts of our country. The profession should rouse itself from this apathy, and warn the community everywhere of the danger, while offering them the means of security. We may be accused of self-interest in urging this measure of precaution; as our own instrumentality may be necessary, and must be compensated where the means exist. But a moment's reflection must convince the most stupid, that it would be much more to our pecuniary interest to attend a protracted case of small pox, than to perform a trifling operation, which is to prevent it. There are, however, many occasions, in which it is necessary to do our duty at the risk of obloquy; and this is one.

But perhaps I have been somewhat unjust to the profession. The people have in many places, and probably, in some degree, in almost all, chosen other guardians of their health, and rejected our offered aid. It has happened to me to become acquainted with one neighborhood, in which small pox has recently prevailed; but not a single case occurred within the circuit of the regular physician's practice. Those families only suffered who had entrusted the care of their health to an empiric, who, for aught I know, may have been ignorant alike of small pox and of vaccination. It is highly probable that many of those who now hear me could give a similar account of their own neighborhoods. The public should take this subject into their hands. Provision should be made, with legislative sanction, for universal vaccination. If the evil were confined exclusively to the negligent individual, the public might possibly have no right to interfere. But whole communities suffer, and government may and ought to step in for their protection. A man is prohibited by law from setting fire to his own house, because a neighbor's may suffer. Which is the greater evil, that our house should burn, or our families perish with small pox? It might be impossible in this country to establish a system of compulsory vaccination; but legislation might go far towards attaining the same end without this obnoxious feature. Time, however, does not permit me to follow this interesting subject in all its ramifications. I must content myself with having introduced it to your notice. If the profession can do nothing more, they can at least raise a warning voice everywhere; and this will be doing much.

I must close with begging you to excuse the length into which I have been drawn in the discussion of the important points that have engaged our attention. I intended to be very brief; but few men, when they have taken their pen in hand, can say to the flowing tide of their thoughts, "thus far shalt thou go, and no further." Allow me, in a few parting words, to thank you warmly for your attention, and to express the hope that our labors, during the present session, may tend to confirm the good that has been done, and to carry us still further onward in the great road of progress; so that, hereafter, the meeting at Detroit may be remembered as one, at which we may all be gratified and proud to have assisted.

At the conclusion of the address, on motion of Dr. Atlee, of Pa.,

*Resolved*, That the thanks of the Association be presented to our late President for the able and interesting parting address he has just delivered, and that he be requestod to present the Committee of Publication a copy, for preservation in our Transactions.

On motion of Dr. Atlee, of Pa.,

*Resolved*, That a committee of three be appointed to inform the President and Vice Presidents elect of their election, and conduct them to their seats.

The President appointed, as such committee, Drs. Atlee, of Pa., Reeves, of Ohio, and Sutton, of Ky.

Upon taking the chair, Dr. Pitcher said:

Although fully aware of my indebtedness, for this distinction, to your observance of a custom equivalent in force to positive law, of selecting your presiding officer, in each successive year, from the State in which the meeting of the Association is held, I feel myself more honored by your partiality, than if I had received the same mark of respect from any other body of men known to the annals of our country.

This sentiment of regard for the body towards which I now hold, by this act of yours, so delicate and interesting a relation, has been inspired by a contemplation of the ideal of the physician, and strengthened by my growing acquaintance with the individuals which compose it.

Being unaccustomed to presiding in deliberative assemblies, I shall throw myself upon the indulgence of the Association, and rely upon the kindness and intelligent co-operation of the individual members for assistance, in performing the duties of the chair.

Whilst thanking you most cordially for this expression of confidence, I can only assure you that such abilities as I possess shall be devoted to the prosperity of the Association and the harmony of its proceedings

On motion of Dr. Gunn, of Mich.,

*Resolved*, That the resolution passed at St. Louis, requiring a majority of the Committee on Publication to be appointed from residents of the place where the meeting is held, be repealed.

Dr. Phelps, of N. Y., offered the following:

*Whereas*, The pleasure and satisfaction of attending the deliberations of this Association would be greatly enhanced, the duties of the secretaries and reporters facilitated, and order at the same time secured, by the observance of two things, to wit: first, that the audience be put in possession of the name and residence of the speaker; and, secondly, that they be enabled distinctly to hear what he has to say; therefore,

*Resolved*, That no one be permitted to address the Association, except he shall have first given his name and residence, which shall be distinctly announced from the chair, and the member be required to go forward and speak from the stand, and not more than ten minutes at one time.

A motion to lay on the table was lost. The resolution was then adopted.

At the request of Dr. Gross, of Ky., his report upon "The Causes that Retard American Medical Literature," was made the special order for Wednesday at 10 o'clock.

Dr. Palmer, of Ill., from the Committee on Prize Essays and Volunteer Communications, submitted the following:

"The Committee on Prize Essays and Volunteer Communications" report, that some months since they issued a card, which was extensively published in the medical journals, setting forth the terms upon which essays intended for prizes would be received; but that the number of papers presented has been but four.

By referring to the past records of the Association, it is found that the numbers received by preceding committees have been, in 1852, sixteen; in 1853, fifteen; in 1854, nine; in 1855, six; and in 1856, four. Your committee beg leave to call attention to this almost regular and quite rapid decrease in the number of essays presented, for the purpose of having the Association consider whether there be not danger that the number which may hereafter be furnished will be so small as to afford insufficient range of comparison and choice to cause the preference shown to be much valued, if, indeed, presentations do not cease altogether, and whether any means should be devised for preventing such a result.

The essays received by your committee have been subjected to a careful examination; and while admitting that they all possess a degree of merit which would render them suggestive and useful, if given to the profession, still, in their opinion, but one manifests that evidence of careful and laborious investigation, that wide scope and rigid accuracy of logical reasoning, that chasteness of expression and artistic skill in the presentation of the subject, to furnish sufficient claim for awarding a prize by this body.

But one prize is therefore awarded. The essay selected for this honor bears the title—"An Essay on the Arterial Circulation."

It is regarded by the committee as possessing the merits just alluded to, and while not wishing to give an unqualified endorsement of all the views which it contains, they regard it as possessing not only interest in its physiological and scientific relations, but also real value in its pathological and practical bearings.

The production has considerable length, and by the fulness with which the views advanced are discussed, it partakes as much of the nature of a treatise as an essay. It has, at least, one quality which Lord Bacon considered necessary to a treatise, as distinguished from an essay,—it required a degree of leisure on the part of the writer, and will require the same on the part of the reader, for him fully to appreciate its value.

The essay bears the motto—"*Una est Veritas.*"

(Signed) A. B. PALMER, Ch'n.
SAMUEL DENTON,
SILAS H. DOUGLASS,
AB'M SAGER,
E. ANDREWS.

On breaking the seal of the accompanying packet, Dr. Henry Hartshorne, of Philadelphia, Pa., was found to be the successful essayist.

The report was accepted.

Dr. Blatchford, of N. Y., from the Committee on "Hydrophobia, and the Connection of the Season of the Year with its Prevalence," read a report thereon. The committee, in conclusion, submitted the following resolution, which was adopted:

*Resolved*, That the Secretary transmit to the Governor of each State a copy of the statistical part of this report, with the respectful request that he would bring the subject before the Legislature of the State over which he presides, that in their wisdom they may devise and unite upon a plan by which the evil may be mitigated, if not removed.

The Committee on Nominations reported in favor of holding the next annual meeting of the Association at Nashville, Tenn.

Dr. Gross, of Ky., moved to strike out "Nashville, Tenn." and insert "Louisville, Ky." He thought Nashville at present difficult of access.

Dr. Geddings, of S. C., and Lindsley, of Tenn., advocated the adoption of the report.

Dr. Gross withdrew his amendment and the report was adopted.

Dr. Wister, of Pa., from the Committee on Publication, made the annual report. It states that the first copies of the Transactions of the last session of the Association were issued on the 10th of November, 1855; that 1,100 copies were printed; that the aggregate expense of printing, illustrating, and binding was $1,922 70; that the distribution of the volume was effected, in every possible instance, by express; that Drs. C. Hooker of Ct., Alden March of Albany, J. L. Atlee of Pa., W. Brodie of Mich., C. B. Gibson of Richmond, E. L. Beadle of N. Y., H. W. Dessaussure of S. C., C. A. Pope of Mo., D. H. Storer of Mass., T. G. Richardson of Ky., J. Moran of R. I., T. Miller of D. C., F. E. B. Hintze of Md., L. P. Bush of Del., Z. Pitcher of Mich., and J. B. Lindsley of Tenn., have rendered essential service to the Association—some in procuring subscriptions to the volume, and all by cordial co-operation in its distribution; that it is important to secure efficient co-operation in every State by the appointment of gentlemen whose duty it shall be to aid in procuring subscriptions for and circulating the transactions; that Connecticut is especially to be commended for her services in this particular; that not a little embarrassment was experienced by the committee in restoring to the list of permanent members the names of those who had been left off by order of the Association for non-payment of assessments; that they had endeavored, however, by careful comparison of the various lists, to supply all omissions; that the committee had been reluctantly obliged to omit from the transactions two valuable reports on epidemic diseases—by Dr. L. H. Anderson, of Ala., and Dr. E. D. Fenner, of New Orleans,—but, as they had not been presented to the Association, and acted on by that body, there was no other alternative; and the following resolution, passed at the last session, should be strictly enforced:

*Resolved*, That, hereafter, beginning with the session of 1856, no report, or other paper, shall be entitled to publication in the volume for for the year in which it shall be presented to the Association, unless it be placed in the hands of the Committee of Publication on or before June 1st.

The report further states that the number of volumes of Transactions now remaining on hand is as follows: of Vol. I. 41, of Vol. II. 9, of Vol. III. 32, of Vol. IV. 7, of Vol. V. 316, of Vol. VI. 66, of Vol. VII. 120, of Vol. VIII. 351; that some of the leading journals abroad have expressed a strong desire to complete their sets, and it rests with the Association to determine whether the missing numbers shall be supplied; that, as only seven complete sets of the Transactions are now in the possession of the Association, the committee recommend that no copy of either of the eight volumes which is necessary to the complete sets now remaining shall be disposed of separately or with any number of volumes short of a complete set.

Dr. Atlee, of Pa., made some remarks upon the report, in the course of which he stated that the Smithsonian Institution had been offered as a permanent place of session for the Association. He concluded by moving that the Committee on Publication preserve five complete sets of the proceedings. Carried.

Dr. Wood, of Philadelphia, moved to refer the nomination of standing committees to the Committee on Nominations. Carried.

The same gentleman made a request, in behalf of Dr. Hamilton, that the committee of which Dr. H. is chairman may be continued for another year, it not being prepared to report at present. Granted.

Dr. Breckenridge, of Ky., stated that the Committee on Medical Literature was ready to report.

The President suggested that the reading of the report follow that of the report of Dr. Gross, which had been made the special order for Wednesday, as 10 A. M.

Dr. Palmer, of Chicago, stated that the Committee on Plan of Organization for State and County Medical Societies was ready to report.

Dr. Pomeroy, of N. Y., moved to reconsider the resolution requiring a member, when speaking, to stand upon the platform, and not to occupy more than ten minutes in his remarks. Lost.

Dr. Smith, of N. J., moved that that portion of the resolution requiring members, when speaking, to take the stand, be rescinded. Carried.

Dr. Atlee, of Pa., moved to refer the prize essay of Dr. Hartshorne on Arterial Circulation, and the report of Dr. Blatchford on Hydrophobia, to the Committee on Publication. Carried.

Dr. Wister, of Pa., the Treasurer, read his annual report. It recommends that the Treasurer be requested, at an early date after the adjournment of the present meeting, to address a circular to each permanent member, announcing the abrogation of the resolution of 1854—making a yearly subscription to the Transactions obligatory—and the consequent restoration to membership of all those dropped from the published list of that year,—advertising, also, the practicability of procuring back numbers of the Transactions, with information as to the cost at which the series of volumes may be rendered complete, or an entire set furnished by the Association.

The account of the Treasurer with the Association is as follows:

DR.

| | |
|---|---|
| To cash paid Dr. John L. Atlee, of Committee on Washington Monument Stone, - - - - - - | $498 70 |
| To cash paid C. B. Norton, for porterage and packing Vol. III., in New York, - - - - - - - - | 8 00 |
| To cash paid J. D. Trask, for Prize Essay, - - - - | 100 00 |
| To cash paid for postage of Secretary, - - - | 2 50 |
| To cash paid D. C. Baxter, for engravings of Vol. VIII, - - | 72 75 |
| To cash paid for postage of Chairman of Publication Committee, - | 4 09 |
| To cash paid Thos. Sinclair & Co.. for lithographs for Vol. VIII, - | 101 20 |
| To cash paid T. R. & P. G. Collins for printing and binding 1,100 copies of Vol. VIII, - - - - - - - - | 1,748 75 |
| To cash paid T. R. & P. G. Collins for binding 25 copies Vol. VIII., and printing notices, - - - - - - - | 4 52 |
| To cash paid H. Barnes for distribution of Vol. VIII., and services as clerk, - - - - - - - | 50 00 |
| To cash paid T. R. & P. G. Collinis for printing notices, - - | 1 25 |
| To cash paid Blanchard & Lea for freight, porterage, boxes, &c., for Vol. VIII, - - - - - - - | 34 99 |
| To cash paid for postage, envelopes, and stationery of Treasurer, - | 6 99 |
| To balance, - - - - - - - | 950 52 |
| | $3,584 26 |

CR.

| | |
|---|---|
| By cash received from Dr. Isaac Wood, being the balance in the Treasury April 30th, 1855, - - - - - - | $1,015 26 |
| By cash received from Dr. Isaac Wood, being the balance in the Treasury of prize essay fund, April 30th, 1855, - - - | 100 00 |
| By cash received from assessment and the sale of Transactions, | 2,150 50 |
| By cash received from Dr. E. L. Beadle for the sale of Transactions, | 12 00 |
| By cash received from Dr. Wm. Brodie for do. - - - | 12 00 |
| By cash received from Dr. A. March for do. - - - | 24 00 |
| By cash received from Messrs. Blanchard & Lea for do. - | 102 50 |
| By cast received from Dr. Chas. Hooker for do. - - | 168 00 |
| | $3,584 26 |

The correctness of this account is certified to by the proper committee.

The report was accepted, and referred to the Committee on Publication.

Dr. McNulty, of the New York Academy of Medicine offered a resolution, that a committee of one from each State be appointed by the Committee on Nominations, to prepare, and report to the Association during the present session, an address to the people of the United States, setting forth the strong claims the medical profession have on their respect, gratitude and confidence.

Dr. McNulty explained the purpose for which he offered the resolution. Many people, he said, had a prejudice against the medical profession for holding to the dignities of their calling, and entertained the idea that the science of medicine was a collection of absurdities and superstitions. He wanted to show clearly that this is not the fact, and, in this view, he thought the address proposed would have a beneficial affect.

Dr. Kittredge moved to amend the resolution by making it read that every member of the Association should take the stump and defend the cause.

After a few other remarks the resolution was withdrawn.

A gentleman, whose name we did not learn, stated that Dr. Wood, of New York, who was then in the meeting, had lately performed an operation in an extraordinary case,—removing a jaw-bone,—and moved that a time be appointed for the Association to examine the part extirpated.

Dr. Wood said he had not with him the article spoken of by the preceding speaker, but would lay it on the desk of the President this morning.

The President read a communication from Dr. Stillé, chairman of of the committee appointed last year to consider the subject of extending the lectures of each chair in medical schools over a period of two years, stating that the views of medical institutions had as yet been imperfectly ascertained, and asking a continuance of the committee. Granted.

Dr. Watson, of N. Y., moved that the Committee on Epidemics meet immediately after the adjourment. Agreed to.

The President read an invitation to the Association to attend the session of the American Association for the Advancement of Science, at Albany, in August next,—at which time, also, the Dudley Observatory will be inaugurated, and an address delivered by Hon. Edward Everett. The invitation was accepted.

The Association then adjourned to meet on Wednesday morning at 9 o'clock.

*May 7th—Morning Session.*

The Association was called to order by the President, at nine o'clock.

The minutes of the previous session were read, corrected and approved.

The names of the delegates who had registered themselves since the last report were read.

The Secretary read communications from the following gentlemen asking an extension of time in which to report upon the subjects named:

Dr. A. J. Semmes, of N. Y.,—" Coroners' Inquests."

Dr. J. Taylor Bradford, of Ky.,—"Treatment of Cholera."

Dr. J. M. Reese, of N. Y.,—"Infant Mortality."

Dr. E. R. Peaslee, of Me.,—"Inflammation, &c."

Dr. J. W. Corson, of N. York,—"The Causes of the impulse of the Heart, and the Agencies which Influence it in Health and Disease."

Dr. Mark Stephenson, of N. Y.,—"The Treatment Best Adapted to Each Variety of Cataract, with the Method of Operation, Place of Election, Time, Age, &c."

Dr. Beech, of Mich.,—"Medical Topography, and Epidemics."

Dr. J. C. Hutchinson, of N. Y.,—"The Anatomy and Histology of the Cervix Uteri."

Referred to Committee on Nominations.

The Secretary announced that he had received the following resolution adopted at the last meeting of the New York State Medical Society:

*Resolved*, That the members of the American Medical Association be invited to attend the semi-centennial celebration of this society, which will occur on the first Tuesday of February, 1857.

The invitation was accepted.

The Secretary read the following communication, dated April 7, 1856, from the Secretary of the Ohio State Medical Society:

Sir—At the annual meeting of this society, held in June last, at Zanesville, Ohio, the following resolutions were adopted, and I was directed to furnish you with a copy of the same:

*Resolved*, That the resolution offered by Dr. Grant, (a member of this society, but not at this or at that time a practitioner of medicine, but a lawyer,) at the last session of this society, viz: "That it is not derogatory to medical dignity, or inconsistent with medical honor, for medical gentlemen to take out a patent-right for surgical or mechanical instruments," was offered at a time when many members had left for their homes, and is not, therefore, the sense of the society.

*Resolved*, That the said resolution is in direct opposition to the code of medical ethics adopted by this society; and, therefore, be it further

*Resolved*, That said resolution, offered by Dr. Grant, and adopted by the society, be and is hereby, rescinded.

The communication was ordered to be placed upon the minutes.

The Secretary read a communication from Dr. Hamilton, of Buffalo, N. Y., transmitting the second part of a report upon Deformities after fracture and dislocations, and asking for a correction of the minutes of last session in regard thereto. Dr. H. also asked that he be permitted to incorporate, in a volume upon the subject he is preparing for publication, that portion of the report already published by the Association.

On motion of Dr. Brodie, of Mich., the minutes were amended.

Dr. Atlee, of Pa., offered a resolution that the request of Dr. H, in regard to the publication of the report, be granted.

Dr. Lindsley, of Tenn., opposed the resolution. A similar request was denied at the session of the Association held at St. Louis.

Dr. Palmer, of Ill., moved to refer the matter to a special committee. Carried.

The President appointed as such committee, Drs. Palmer, of Ill., Atlee, of Pa., and Hills, of Ohio.

Dr. Gunn, of Michigan, moved that those gentlemen from Canada, who are here by general invitation, be admitted in a body, and be requested to take seats on the platform during this morning's session. Carried.

Several gentlemen complied with the invitation:

Dr. Sutton, of Ky., offered a resolution that 1,000 copies of the address of the late President, Dr. Wood, be published. Adopted.

On motion of Dr. J. Lindsley, of Tenn.,

*Resolved,* That a committee of three be appointed by the Chair, to prepare a suitable minute in reference to the death of our late Secretary, Dr. P. C. Gooch, of Richmond, Va., who fell a martyr while contending with the pestilence in Norfolk, in 1855.

The President appointed as such committee, Drs. Lindsley, of Tenn., Thomson, of Del., and Mendenhall, of Ohio.

Dr. Gross, of Ky., from committee appointed the day previous, reported the following preamble and resolutions relative to the death of Dr. J. C. Warren, of Boston:

*Whereas,* It has pleased Almighty God to remove from the scene of his earthly labors our late fellow-member, Dr. John C. Warren, of Boston, formerly President of this Association, and for many years Professor of Anatomy and Surgery in Harvard University;

*And whereas,* It is just and proper that, when a great and good man dies, his memory should be cherished by his fellow-citizens, and transmitted unimpaired to posterity for the encouragement of future ages; therefore,

*Resolved,* That this Association has learned with profound regret the news of an event which has deprived the American medical profession of one of its oldest, most useful, and most illustrious members—American surgery one of its greatest ornaments—science one of its best friends—and humanity one of its noblest benefactors.

*Resolved,* That the life of Dr. John C. Warren affords an example of a man who, notwithstanding the possession of ample riches, devoted himself, heart and soul, for upwards of half a century, to the cultivation and advancement of his profession, and to the good of the human race.

*Resolved,* That this Association deeply sympathizes with the family of Dr. Warren in their bereavement, and that the Secretary be requested to transmit to them a copy of these proceedings.

The preamble and resolutions were adopted and referred to the Committee on Publication.

Dr. Gross, of Ky., read a report on "The Causes which Impede the Progress of American Medical Literature." In conclusion, he submitted the following resolutions:

*Resolved,* That this Association earnestly and respectfully recommends: 1st. The universal adoption, whenever practicable, by our schools, of American works, as text books for their pupils. 2d. The discontinuance of the practice of editing foreign writings. 3d. A more independent course of the medical periodical press towards foreign productions, and a more liberal one towards American; and, 4th. A better and more efficient employment of the facts which are continually furnished hy our public institutions, for the elucidation of the nature of diseases and accidents, and, indirectly, for the formation of an original, a vigorous, and an independent national medical literature.

*Resolved*, That we venerate the writings of the great medical men, past and present, of our country, and that we consider them as an important element of our national medical literature.

*Resolved*, That we shall always hail with pleasure any useful or valuable work emanating from the European press, and that we shall always extend to them a cordial welcome, as books of reference, to acquaint us with the progress of legitimate medicine abroad, and to enlighten us in regard to any new facts of which they may be the repositories.

Dr. Phelps, of New York, moved that the report and resolutions, as a whole, be adopted.

At the suggestion of a member, the question was divided. The report was adopted.

Upon the reading of the first resolution, a member proposed to substitute "just" for "liberal." Dr. Gross accepted the amendment.

Dr. Palmer, of Ill., wished to understand the meaning of the word "practicable," as employed in the resolutions. If it meant that an inferior work by an American author was to be used in our medical schools, in preference to a superior one, treating of the same subject, by a foreign author, he was decidedly opposed to the resolution. If, when the American work is equal or superior to the foreign one, it is to be used, then he had no objection. He alluded to works by eminent English and French authors.

Dr. Gross explained. One of the works alluded to by Dr. P. must of necessity be used in our medical institutions of learning, as there is no work by an American author on the same subject. Foreign works should be used as books of reference, and American books, "when practicable," as text books.

Dr. Yandell, of Ky., moved that the resolutions be made the special order for Thursday morning. Lost.

Dr. Cobb, of N. Y., was opposed to the resolutions. If adopted and sent out to the world, they savor too much of know-nothingism to make them palatable. (Sensation.)

Dr. Leidy, of Pa , was in favor of leaving to teachers of medicine the selection of their own text books.

Dr. Davis understood there was another report touching upon the subject—that upon "American Medical Literature," by Dr. Breckenridge, of Ky. He moved to lay the resolutions upon the table until that report was read. Carried.

The Secretary read a communication from Dr. P. A. Jewett, of Conn., chairman of the Committee to Procure Memoirs of the Eminent and Worthy Dead. Referred to Committee on Nominations.

Dr. Breckenridge, of Ky., read a report upon American Medical Literature.

On motion of Dr. Hooker, it was accepted and referred to the Committee on Publication.

The Association then adjourned on Thursday morning at 9 o'clock.

*Thursday, May 8th—Morning Session.*

The Association was called to order by the President at 9 o'clock.

The minutes were read, corrected and approved.

A communication from Dr. Wroth, of Md., relative to a report upon the Medical Topography of the eastern shore of Maryland, and one from Dr. Thomson, of Ky., relative to a report on "Chloroform," were referred to the Committee on Nominations.

The Secretary read a letter from E. S. Lesmoines, of St. Louis, enclosing an autograph letter from M. Dubois.

The Secretary read a communication from J. C. Holmes, Esq., the Secretary of the Michigan State Agricultural Society, presenting to the Association twenty-five copies of the Transactions of the Society for 1853, and also the same number of Transactions for 1854.

Dr. Brodie, of Mich., moved that the thanks of the Association be returned therefor, and that one copy be presented to each State represented. Carried.

On motion, Dr. Maggugin, of Iowa, was appointed a member from that State of the Committee on Nominations.

On motion of Dr. Atlee, of Pa.,

*Resolved,* That the President shall be authorized annually to appoint delegates to represent this Association, at the Meetings of the British Association, the American Medical Society of Paris, and such other scientific bodies in Europe, as may be affiliated with us. Adopted.

Dr. Gluck, of New York, offered the following:

*Whereas,* During the present year a medical congress is to be held in Europe; therefore

*Resolved,* That the American Medical Association send to that Congress four delegates, representing the four sections of the Union.

Dr. Davis, of Ill., thought it might be necessary and proper to send a greater number than four. He moved to lay the resolution on the table. Carried.

Dr. Clendenin offered the following:

*Resolved,* That a committee of one be appointed, for a period of three years, with instructions to report progress at each annual meeting of this Society, to investigate the etiology and pathology of epidemic cholera, and that said committee be allowed to add any other members to the same which they think may be necessary to further the objects of the appointment.

On motion, referred to the Committee on Nominations.

On motion of Dr. Mendenhall, of Ohio.

*Resolved,* That the Secretary be instructed to strike the name of C. H. Cleveland from the list of permanent members of this Association.

On motion of Dr. Atlee, of Pa.,

*Resolved,* That the name of James R. McClintock be stricken from the list of permanent members.

These expelled members were accused by the movers of the resolutions of having retrograded into quackery.

On motion of Dr. Bissell, of New York,

*Resolved,* That this Association has learned with deep regret the death of one of its members, Dr. Theodore Romeyn Beck, of Albany, N. Y., whose whole life has been devoted to the attainment and promotion of medicine and general science, and that we do herebv express our high appreciation of the excellencies of his character, distinguished by its simplicity, integrity, and firmness of purpose, and by the extent and variety of his acquirements in medical as well as in almost every other department of science.

*Resolved,* That the above resolution be referred to the committee to procure memorials of the eminent and worthy dead, and that they be requested to procure a memoir of the late Dr. Beck, to be published in the Transactions of the Association.

Dr. Bloodgood, of Ill., offered the following:

*Resolved,* That the constitution of this Association be so amended as that hereafter the President shall be elected by ballot, and shall not be a resident of the State in which he is elected.

On motion of Dr. Watson, of N. Y., laid on the table.

Dr. Wister, of Pa., offered the following, which was adopted.

*Resolved,* That the invitation to gentlemen of the medical profession of Canada, extended to them by the American Medical Association at its session in Philadelphia, be renewed for the meeting of Nashville, Tenn.; and that this Association may be safe from the introduction of unsuitable persons, it is recommended that gentlemen presenting themselves from the Province of Canada should be provided with a letter of introduction to this Association from one of the following gentlemen: Drs. Tarquand, A. Scott, Woodstock, Canada; Drs. Hodder, Bethune, Richardson, Bonell, Haswell, Widmer, Beaumont, Herrick, of Toronto; Drs. O'Reilly, Craiggie, Duggan, of Hamilton; Dr. Sampson, of Kingston.

Dr. Gunn, of the Committee of Arrangements, stated, that as several delegates from the east had manifested a desire to go to Buffalo on the magnificent steamer *Western World*, of the Michigan Central Railroad line, the agent had kindly acceded to a request to sail her for Buffalo on Friday, (in advance of her regular time), if a specified number would take passage.

Dr. Phelps, of New York, offered the following:

*Whereas,* It has pleased an All-Wise, but Inscrutable Providence to visit the city of Norfolk, Va., and vicinity, with a desolating pestilence, equal, or surpassing, any recorded in ancient or modern times, and by which, in a few weeks, forty physicians, either residents, or those from abroad, who had promptly rushed to the rescue, among the number of whom was our late Secretary and associate, Dr. Gooch, of Richmond, had been swept away, therefore

*Resolved,* That such an instance of signal and unflinching devotion to the cause of science and of humanity demands at the hands of this national Association a passing expression of their high admiration of this, another memorable instance of the unparalleled sacrifices of the profession to the interests of the healing art and of the race.

*Resolved*, That this minute be incorporated in our Transactions. Adopted.

On motion of Dr. Palmer, of Ill., Rev. Samuel A. McCoskry, Episcopal Bishop of this diocese, was invited to a seat upon the platform.

The like courtesy was extended to Dr. Mussey, formerly President of the Association.

Dr. Stocker, of Pa., offered the following amendments to the constitution:

Amend article 3 so that it shall read: "Article 3. The regular meetings of the Association shall be held annually, and commence on the first Tuesday of May. The Association shall meet biennially in the city of——. The place of meeting for the intermediate year shall be determined by a vote of the Association."

Amend article 4 by providing for one permanent and two assistant secretaries, and also specifying the duties, &c. of each.

Laid on the table under the rule.

Dr. Dorsey, of Ohio, offered the following:

*Resolved*, That in May, 1858, and every third year thereafter, this Association meet at Washington city, and that the present officers be requested to correspond with the Board of Managers of the Smithsonian Institute, in regard to furnishing necessary rooms for the keeping of the archives of the Association.

Laid on the table under the rule.

On motion of Dr. Sheets,

*Resolved*, That it is derogatory to the dignity of the medical profession to notice the works of irregular practitioners in our medical periodicals.

Dr. Frost, of S. C., objected to the introduction of resolutions. He thought it irregular.—Reports were the order.

Dr. Davis, of Ill., moved that reports be made the special order. Carried.

Dr. Watson, of N. Y., moved to reconsider the last vote, in order to take up the resolutions attached to the report of Dr. Gross, of Ky., upon the "Causes which Retard American Medical Literature." Carried.

The resolutions were taken up. The question being upon their adoption,

Dr. Gross read extracts from his report, explained the intent of the resolutions, insisted upon their necessity, and advocated their adoption.

Dr. Davis, of Ill., was opposed to adopting any report. There were now before the Association two reports, [the one by Dr. Gross, of Ky., and one by Dr. Breckenridge, of Ky.,] presenting directly adverse views. He thought both should be accepted and referred to the proper committee—nothing more.

Dr. Breckenridge, of Ky., said the point at issue is—whether the Association will favor the sectionalism or the universality of medicine.

If Dr. Gross' report and resolutions are adopted we decided in favor of the former.

Dr. Cobb, of N. Y., foresaw the difficulty that would arise in adopting Dr. Gross' report the day previous.

Dr. Watson, of N. Y., moved to reconsider the vote by which the report was adopted.—Carried.

He then moved that the report be accepted.—Carried.

On motion of Dr. Atlee, of Pa., the report and resolutions of Dr. Gross, and the report of Dr. Breckenridge, upon "American Medical Literature," were referred to the Committee of Publication

Dr. Palmer, of Ill., from special committee to which was referred the communication of Dr. Hamilton, reported the following resolution, which was adopted:

*Resolved*, That leave be granted to Dr. F. H. Hamilton to make use of the materials of his report on "Deformities after Fractures," which is in course of publication by this association, in his anticipated work upon "Fractures and Dislocations."

Dr. A. B. Palmer, Professor in the Michigan University, from the Committee on Plans of Organization for State and County Medical Societies, presented a lengthened and able report, containing numerous useful suggestions, with outlines for the proper organization of local societies, and a series of resolutions in accordance with the views enforced in the report. Accepted, and referred to the Committee on Publication.

On motion, the resolutions were temporarily laid on the table for further action by the Convention.

Dr. Davis, of Illinois, chairman of special committee, reported on "The Changes in the Composition and Properties of the Milk of the Human Female, produced by Menstruation and Pregnancy," in a paper containing numerous valuable details of much interest to the profession and the public, obtained by careful examination and comparison, and showing conclusively the ill effects of lactation, especially during the latter of the periods referred to. Accepted, and referred to Committee on Publication.

Dr. Chas. Q. Chandler, of Missouri, who was to report on "Malignant Periodic Fevers," submitted, as a substitute, through Secretary Brodie, a paper on "Sulphate of Cinchona," which was received as a "voluntary contribution," and referred to a special committee.

Dr. Johnson, of Chicago, asked further time to report on "Excretions, &c." Referred to Committee on Nominations.

Dr. J. M. Newman, of Buffalo, from Committee on "the Sanitary Police of Cities," presented an elaborate report, embracing details of the various estimated causes of disease in cities, as compared with rural localities, together with numerous valuable statistics of mortality in the largest cities of Europe and the Union, of which the Doctor, at the request of the Association, gave a brief, verbal abstract. The report evidently embodies a vast mass of useful information, with deductions from it that city life is inimical to health and longevity, and arguments enforcing the urgent necessity for ameliorating the sanitary condition of the populous localities of cities and large towns. Of diseases arising from impure air and insufficient ventilation, classed under the term "zimotic," the report stated that, in 1850, 40 per cent of all the deaths in the various cities were of that nature. The report also embodied details of the loss of life from cholera, small pox, &c., giving startling expositions of danger from these sources, and recommends the enactment of laws for compulsory ventilation and cleanliness, as well as for vaccination, &c. Accepted and referred to Committee on Publication.

The President here requested such delegates as would prefer to take passage, on their return, on the Michigan Central Railroad Company's steamer Western World for Buffalo, which leaves to-day at 12 M., to signify their wishes.

Adjourned to 2 P. M.

*May 8th—Afternoon Session.*

The Association met at 2 o'clock.

Dr. Frost, of Charleston, S. C., offered the following resolution, which was adopted:—

*Resolved,* That the thanks of this Association are due to the retiring officers for the zealous and efficient manner in which their duties have been performed; to our late President, for the courtesy and ability with which he has presided over our deliberations; to all the officers, for their attention to the laborious duties of their stations—not excepting our Committee of Publication, to whom we must feel indebted for the satisfactory form in which the volume of the Transactions appears.

Dr. A. J. Fuller, of Me., chairman of the Committee on the Best Treatment of Cholera Infantum, read a report thereon, in which he stated that the pathology of the disease was little understood, and that physicians should interchange views on the subject.

The report was accepted and referred to the Committee on Publication.

Dr. Green, of N. Y., chairman of the Committee on the Use and Effects of Application of Nitrate of Silver to the Throat, read a report thereon. He asserted that great benefits had been derived from topical medication in thoracic disease,—tuberculosis, bronchitis, &c. The report was accepted and referred to the Committee on Publication.

Dr. Flint, of Louisville, Chairman of the Committee on the Best Mode of Rendering the Medical Patronage of the National Government Tributary to the Honor and Improvement of the Profession, read a report thereon. He denounced the granting of patents by the United States government to "quack medicines,"—stating, however, that it appears, from a letter written by the present Commissioner of Patents, that the practice of the Office has been to discourage such a use of its functions, and that, during the past 15 years, but four or five such patents have been granted, although from twenty to thirty applications therefor have been made per year. The credit of sanitary improvements, Dr. F. said, was not due to individuals, but to medical science. Such improvements are never discoveries or revelations, but inductions. The United States government should aid the great cause of medical science by making appropriations for the publication of the Transactions of the National

Association, and by paying prizes for the best essays on subjects selected by that Association. The report was accepted and referred to the Committee on Publication.

The Committee on Nominations made the following report:—

The Nominating Committee beg leave to make the following report:

For Chairmen of Special Committees for 1857:—

Dr. E. R. Peaslee, of Brunswick, Me., on Inflammation, its Pathology and its Relation to the Recuperative Process.

Dr. J. C. Hutchinson, of Brooklyn, N. Y., and Charles E. Isaacs, of New York city, on the Anatomy and Histology of the Cervix Uteri.

Dr. J. Taylor Bradford, of Augusta, Ky., on the treatment of Cholera.

Dr. Mark Stephenson, of N. Y., on the Treatment Best Adapted to Each Variety of Cataract, with the Method of Operation, Place of Election, Time, Age, &c.

Dr. J. W. Corson, of N. Y., on the Causes of the Impulse of the Heart, and the Agencies which Influence it in Health and Disease.

Dr. D. Meredith Reese, of N. Y., on the Causes of Infant Mortality in Large Cities, the Source of its Increase, and the Means for its Diminution.

Dr. J. Foster Jenkins, of Yonkers, N. Y., on Spontaneous Umbilical Hæmorrhage of the Newly Born.

Dr. Henry Carpenter, of Lancaster, Pa., on the Use of Instruments in Obstetrical Practice.

Dr. Alex. J. Semmes, of Washington, D. C., on the Measures to be Adopted to Remedy the Evils Existing in the Present Mode of Holding Coroners' Inquests.

Dr. J. Marion Sims, of New York city, on the Treatment of the Results of Obstructed Labor.

Dr. J. B. Flint, of Louisville, Ky., on the True Position and Value of Operative Surgery as a Therapeutic Agent.

Dr. G. Volney Dorsey, of Piqua, Ohio, on the Causes and Cure of Indigestion, especially in Relation to the Therapeutic Indications to be derived from the chemical composition of the Deposits in the Urine.

Dr. C. B. Coventry, of Utica, N. Y., on the Medical Jurisprudence of Insanity, and the Testimony of Skilled Witnesses in Courts of Justice.

Dr. Jos. Leidy, of Philadelphia, Pa., on Human, Animal, and Vegetable Parasites.

Dr. M. D. Darnall, of Bainbridge, Ind., on the Value of a Strict Attention to Position in the Treatment of Diseases of the Abdomen.

Dr. George Sutton, of Aurora, Ind., on Milk Sickness.

Dr. Clark J. Pease, of Janesville, Wis., on the Blending and Conversion of the Types of Fever.

Dr. B. S. Woodsworth, of Fort Wayne, Ind., on the Best Substitute for Cinchona and its Preparations in the Treatment of Intermittent Fever and Malarious Neuralgia.

Dr. Franklin Hinkle, of Marietta, Pa., on the Use of Cinchona in Malarious Diseases.

Dr. Henry V. Campbell, of Augusta, Ga., on the Nervous System in Febrile Diseases.

Dr. John Neill, of Philadelphia, Penn., on the Laws Governing the Absorption and Deposit of Bone.

Dr. John W. Greene, of N. Y. city, on the Intimate Effects of Certain Toxicological Agents in the Animal Tissues and Fluids.

Dr. George Suckley, U. S. A., on the Medical Topography and Fauna of Washington Territory.

Dr. Jas. Cooper, of Hoboken, N. J., on the Flora of Washington and Oregon Territories.

Dr. Chas. E. Isaacs, of N. Y., on the Intimate Structure and the Pathology of the Kidney.

Dr. Israel Moses, of New York city, on the Diseases Incidental to Europeans from temperate Climates in their Transition through Central America.

Dr. T. W. Gordon, of Georgetown, Brown County, O., on the Etiology and Pathology of Epidemic Cholera, to be continued three years, and with power to add any other members.

Dr. H. A. Johnson, of Chicago, on the Excretions as an Index to the Organic Changes going on in the System.

Dr. D. D. Thomson, of Louisville, on the Remedial Effects of Chloroform.

STANDING COMMITTEES—*Committee on Publication*—Drs. Francis G. Smith, of Pa., Chairman; Caspar Wister, of Pa.; Samuel L. Hollingsworth, of Pa.; Samuel Lewis, of Pa.; H. F. Askew, of Del.; Wm. Brodie, of Mich.; R. C. Foster, of Tenn.

*Committee on Prize Essays*—Drs. Wm. K. Bowling, of Tenn., Chairman; E. B. Haskins, of Tenn.; Thomas Lipscomb, of Tenn.; A. H. Buchanan, of Tenn.; B. W. Avent, of Tenn.; W. A. Cheatham, of Tenn.; Paul F. Eve, of Tenn.

*Committee of Arrangements*—Drs. C. K. Winston, of Tenn., Chairman; Ira Conwell, of Tenn.; William D. Haggart, of Tenn.; J. L. C. Johnson, of Tenn.; F. A. Ramsey, of Tenn.; Geo. Grant, of Tenn.; J. B. Lindsley, of Tenn.

To fill vacancies in the Committee on Medical Topography and Epidemics :—

*New Hampshire*—Dr. V. P. Fitch, of Amherst.

*California*—Dr. Robert Murray, of Fort Miller.

To fill vacancies in the Committee upon a Uniform System of Registration of Marriages, Births and Deaths :—

*Vermont*—Dr. Adrian T. Woodward, of Castleton.

*Connecticut*—Dr. Wm. B. Casey, of Middletown.

*Virginia*—Dr. R. W. Haxall, of Richmond.

*California*—Dr. Arthur R. Stout, of San Francisco.

They recommend the continuance of the "Committee to Procure Memorials of the Eminent and Worthy Dead," and that the report, as far as prepared, be referred to the Committee on Publication.

STANDING COMMITTEES.—*On Medical Education*—Drs. E. Geddings, of S. C., Chairman; C. W. Le Boutillier, of Minnesota; G. F. Mitchell, of Ohio; S. W. Clanton, of Ala.; S. W. Butler, of N. J.

*On Medical Literature*—Drs. R. Hills, of Ohio, Chairman; D. W.

Yandell, of Ky. ; R. R. Porter, of Del.; H. A. Johnson, of Ill. ; Charles E. Swan, of Maine.

The President stated that Dr. Anderson, of Ala., Chairman of Committee on Medical Education, had sent in his report. It was accepted and referred to the Committee on Publication.

A report from Dr. Worth, of Md., on the Medical Topography and Epidemics of the Eastern Shore of Maryland, was accepted and referred to the Committee on Publication.

A report from Dr. Cain, of S. C., on the Epidemic of Yellow Fever in Charleston, in 1854, was accepted and referred to the Committee on Publication.

A report from Dr. Fenner, of La., on the Medical Topography and Epidemics of Louisiana, was accepted and referred to the Committee on Publication.

Secretary Brodie stated, that he had received a letter from Dr. Dillard, U. S. N., appointed on the Committee on Medical Topography and Epidemics, saying that he could not act, in consequence of having received no appointment as delegate to the Association from the Surgeon General.

Dr. Gunn, of Michigan, said three communications had been handed to the Committee of Arrangements by the Secretaries, which they (the Committee) had not time to examine. He asked that a special committee be appointed to report on volunteer communications.

Dr. Palmer, of Ill., offered the following, which was adopted :—

*Resolved*, That the volunteer communications in the hands of the Committee of Arrangements be referred, with all other such communications, to a special committee to be appointed by the Chair, residing at the place of publication of the Transactions; and if, in their judgment, the papers are worthy, they be referred by them to the Committee on Publication, to go into the Transactions of the Association.

The President appointed as such committee, Drs. A. Stillè, S. Jackson and J. B. Biddle.

The authors and titles of the volunteer communications were announced by Secretary Brodie as follows :—

By Dr. C. J. Chandler, of Rocheport, Mo., on Sulph. Cinchona in Periodic Diseases.

By Dr. Isidor Gluck, of New York, on Formation of Gun Shot Wounds, &c.

By Dr. G. P. Flachenberg, on an Improved Method of Applying Compression to the Scrotum.

A member of the Committee on a Uniform System of Registration of Marriages, Births and Deaths, stated that they were unable to make a report at present, in consequence of the death of their Chairman, Dr. Wilson, of Conn.

The Committee on Medical Literature, for 1855, was continued for another year.

Dr. Neill, of Phil., offered the following resolution :

" That no paper detailing operations or cures be brought before the Association or entered upon its mlnutes, except through a standing or special committee appointed upon the subject."

Dr. Wood, of N. Y., felt wounded at the resolution, and the mover was requested to withdraw it.

Dr. Neill agreed to withdraw the resolution, provided the minutes were corrected.

Dr. Wood's case was expunged from the minutes, and the resolution was withdrawn.

Dr. Gross, of Louisville, tendered, in behalf of the medical profession and the citizens of Louisville, an invitation to the Association to meet in that city in May, 1857. Placed on file.

Dr. Dorsey, of Ohio, offered the following resolution, which was adopted :—

*Resolved*, by the American Medical Association, That the Committee of the Etiology and Pathology of Cholera be instructed to memorialize the Congress of the United States, requesting that honorable body to grant every necessary assistance which can or will promote the objects for which the Committee has been appointed.

Secretary Brodie read a communication from the Royal Medical and Chirurgical Society of England, thanking the American Medical Association for their present of the eighth volume of their Transactions. Ordered placed on file.

Dr. Wister, of Pa., offered the following, which was adopted :—

*Resolved*, That a committee of three be appointed by the President, to correspond with the proper officer of the Smithsonian Institute, inquiring into the possibility of procuring a chamber in that institution, for the uses of this Association.

The President appointed as such committee, Drs. Wister, of Pa., Hale, of Washington, and Neill, of Pa.

*May 9th—Morning Session.*

The President called the Association to order. The Secretary read the minutes of the preceding Session, which were corrected and approved.

Dr. Palmer, of Ill., moved that Dr. Richard Coolidge, of D. C., be substituted in place of Dr. Finley, on the Committee to report on Epidemics. Carried.

Dr. Atlee, of Pa., offered the following :—

*Resolved*, That all voluntary communications, hereafter presented to the Association, shall be referred to a special committee of ——, to be appointed by the President, on the first annual meeting, whose duty it shall be to examine such communications, and report upon the propriety of their presentation and reference to the Committee on Publication. Carried.

Dr. J. B. Lindsley, from the Committee appointed to prepare a suitable minute, having reference to the death of Dr. P. C. Gooch, late Secretary of the Association, begged leave to report the following preamble and resolutions :—

Whereas, the exhibition of high courage and self-sacrificing devotion to the good of others, is ever honorable to a profession by whose members it is manifested, and worthy of remembrance and emulation :

*Resolved*, That in the death of Dr. P. C. Gooch, of Richmond, Va., who nobly volunteered his services during the pestilence at Norfolk, we recognize a loss to this Association, the profession, and the country. His arduous and successful labors as Secretary of the meeting at Charleston and Richmond, merited the regard of this Association; the zeal, ability, and industry manifested by him as the founder and editor of the Stethoscope—the first medical periodical established in Virginia,—showed his devotion to the cause of medical progress and activity, and the manner of his death, gave signal evidence that he was one of whom his country might well be proud.

*Resolved*, That a copy of these resolutions be transmitted by the Secretary to the relatives of the late Dr. Gooch. Carried.

Dr. Palmer, of Chicago, moved

That the above resolution, together with the suggestions in the report of the Committee on Prize Essays, as to whether any means can be devised to cause an increase of the number of Essays presented, be referred to a Special Committee, of which Dr. Leidy, of Pa., shall be Chairman, to report to the next meeting of the Association. Carried. Drs. G. B. Wood and F. G. Smith, of Philada., were put upon said Committee.

Dr. Samuel Denton, of Michigan, offered the following:—

*Resolved*, That a committee of three be appointed whose duty it shall be to enlist some enterprising publisher, and aid in collecting and arranging material for an American Medical Directory. Laid on the table.

Dr. Smith, of Pa., moved that a special committee be appointed to report at the next meeting of this Association, a classification of those diseases, which involve a derangement of the mental manifestations. Carried, and leave given the mover to appoint the committee, he being Chairman.

The Secretary read an invitation from Dr. Childs, of Boston, inviting the members of the Association to meet with the Massachusetts Medical Society, on the last Wednesday of May. Accepted.

Dr. Atlee, of Pa., moved that a copy of the Association's Transactions be sent to the Epidemological Society, at London. Carried.

Dr. James L. Phelps, presented a resolution with several preambles, on the subject that "Medical Ethics, as a branch of general ethics, must rest on the basis of religion and morality."

Dr. Gunn, of Mich., moved that any new Medical Society, not heretofore represented in this Association, be required to transmit to this Secretary, with the credentials of its delegates, the evidence of its existence, capacity and good standing. Carried.

Dr. McGrugan moved that a special committee be appointed to report on the subject of "Stomatitis Matema." Carried.

Dr. Bailey moved that Dr. N. S. Davis be requested to continue his observations on the subject of the changes produced in the composition and qualities of milk by pregnancy and menstruation; also, the best substitute for the mother's milk when weaning becomes necessary before the child is eighteen months old, and report at the next meeting of the Association. Carried.

Dr. Atlee, of Pa., moved that the thanks of the Association be tendered to all railroad companies who had furnished members with passes to this convention. Carried.

Dr. Palmer, of Ill., called for some disposition to be made of the resolutions appended to his report on the organization of Medical Societies.

On motion of Dr. Atlee, they were referred with the report for publication.

Dr. Palmer, of Ill., moved that the Association tender a vote of thanks to the press of Detroit for the interest and attention given to their session in this city. Carried.

Dr. Palmer, of Ill., moved that the Committee on Registrations have leave now to present a partial report, which is hereby referred to the Committee of Publication. Carried.

Dr. Leidy, of Penn., offered the following:—

*Whereas,* It is the object of this Association in the award of its prizes for communications on subjects appertaining to medical science, to encourage the progress of the latter, and as this result cannot be better attained than through original investigation and discovery,

*Resolved,* That hereafter an annual prize of —— be awarded for the best memoir or essay, founded on original investigations of the author, and in case of no memoir or essay being presented worthy of such award, the prize money to be appropriated towards the expense of publishing and illustrating such memoirs as may be subsequently deemed worthy of an award. Carried.

On motion the Association then adjourned to meet at Nashville, Tenn. on the first Tuesday of May, 1857.

